# deconstructSigs: delineating mutational processes in single tumors distinguishes DNA repair deficiencies and patterns of carcinoma evolution

**DOI:** 10.1186/s13059-016-0893-4

**Published:** 2016-02-22

**Authors:** Rachel Rosenthal, Nicholas McGranahan, Javier Herrero, Barry S. Taylor, Charles Swanton

**Affiliations:** UCL Cancer Institute, CRUK Lung Cancer Centre of Excellence, Paul O’Gorman Building, Huntley Street, London, WC1E 6DD UK; The Francis Crick Institute, 44 Lincoln’s Inn Fields, London, WC2A 3LY UK; Centre for Mathematics and Physics in the Life Sciences and Experimental Biology (CoMPLEX), University College London, London, WC1E 6BT UK; UCL Cancer Institute, Bill Lyons Informatics Centre, Paul O’Gorman Building, Huntley Street, London, WC1E 6DD UK; Human Oncology and Pathogenesis Program, Memorial Sloan Kettering Cancer Center, New York, NY USA; Department of Epidemiology and Biostatistics, Memorial Sloan Kettering Cancer Center, New York, NY USA; Center for Molecular Oncology, Memorial Sloan Kettering Cancer Center, New York, NY USA

**Keywords:** Mutational signatures, Esophageal carcinoma, APOBEC, Single samples

## Abstract

**Background:**

Analysis of somatic mutations provides insight into the mutational processes that have shaped the cancer genome, but such analysis currently requires large cohorts. We develop deconstructSigs, which allows the identification of mutational signatures within a single tumor sample.

**Results:**

Application of deconstructSigs identifies samples with DNA repair deficiencies and reveals distinct and dynamic mutational processes molding the cancer genome in esophageal adenocarcinoma compared to squamous cell carcinomas.

**Conclusions:**

deconstructSigs confers the ability to define mutational processes driven by environmental exposures, DNA repair abnormalities, and mutagenic processes in individual tumors with implications for precision cancer medicine.

**Electronic supplementary material:**

The online version of this article (doi:10.1186/s13059-016-0893-4) contains supplementary material, which is available to authorized users.

## Background

The set of somatic mutations observed in a tumor reflects the varied mutational processes that have been active during its life history, providing insights into the routes taken to carcinogenesis. Exogenous mutagens, such as tobacco smoke and ultraviolet light, and endogenous processes, such as APOBEC enzymatic family functional activity or DNA mismatch repair deficiency, result in characteristic patterns of mutation [1, 2]. Thus, through studying the full landscape of mutations present in a tumor and identifying the genomic footprint of mutational signatures that have contributed to them, processes molding the cancer genome during evolution can be revealed and individual therapeutic strategies considered if distinct DNA repair defects are identified [3]. Analysis of mutational signatures has the potential to reveal previously unknown mutagens and occult environmental exposures, such as herbal supplements containing aristolochic acid [3].

Recently, Alexandrov and colleagues developed an algorithm using non-negative matrix factorization (NMF) and model selection to extract the signatures of mutational processes present in a catalog of cancer genomes [4]. Each extracted signature is characterized by the fraction of mutations found in each of the 96 trinucleotide contexts. Additional mutation features such as the presence of indels, dinucleotide mutations, or transcriptional strand bias could also be incorporated into the definition of a mutational signature.

Their published Wellcome Trust Sanger Institute (WTSI) Mutational Signature Framework offers an elegant approach to first identify the signatures of mutational processes present in a set of tumor samples and then apply those signatures to the samples to determine the contribution of each mutational process to each individual sample. However, in order to accurately deconvolute signatures, the number of tumor samples available must be sufficiently large. In simulations, Alexandrov et al. found that at least 200 whole genome samples were required to determine the signatures of 20 mutational processes [5]. With exome sequencing covering only ~1 % of the human genome, resulting in fewer mutations identified, they estimated that it would take thousands of samples to extract the majority of mutational processes that have been functional during tumor life histories.

Using their framework, Alexandrov et al. analyzed approximately five million mutations from over 7000 cancer genomes and exomes to identify a set of 21 signatures found to be present across 30 tumor types [4]. About half of these signatures could be attributed to known mutational processes, such as tobacco smoke, exposure to ultraviolet light, activity of the APOBEC family of cytidine deaminases, DNA mismatch repair deficiency, or mutations in *POLE*. Many signatures, corresponding to the activity of both known and unknown mutational processes, were found across multiple tumor types. Due to the ubiquitous nature of many of the signatures found, there has been interest in quantifying their presence and prevalence in additional tumor samples. However, this is not always possible under the current mutational framework.

In order to address this challenge, we present a method to determine the contributions of each mutational process from a set of published signatures in a single tumor sample.

## Implementation

### Overview of the software

The deconstructSigs approach determines the linear combination of pre-defined signatures that most accurately reconstructs the mutational profile of a single tumor sample. It uses a multiple linear regression model with the caveat that any coefficient must be greater than 0, as negative contributions make no biological sense. The deconstructSigs package is an extension for R, a free programming language and software environment widely used for statistical computing and graphics. This package relies on the Bioconductor library BS.genome.Hsapiens.UCSC.hg19 [6] to acquire mutational context information. It also uses reshape2 [7] for plotting. The R package is publicly available on the CRAN webpage: https://cran.r-project.org/. A detailed README file is also available complete with examples of how to use the package.

### Basic usage

The most basic initial input to the deconstructSigs package consists of a data frame containing the mutational data for a tumor sample set. This structure must contain the genomic position and base change for each mutation, as well as a sample identifier. Using the command “mut.to.sigs.input”, as shown below, the mutational data for one to many tumors is converted to an *n*-row and 96-columns data frame where *n* is the number of unique samples present.

sigs.input < - mut.to.sigs.input(mut.ref = sample.mut.ref, sample.id = "Sample", chr = "chr", pos = "pos", ref = "ref", alt = "alt")

The input data frame *T* is generated by calculating the fraction of mutations found in each of the possible 96 trinucleotide contexts for each tumor sample. By default, no additional normalization is performed. However, when *T* contains only the counts of each mutation in each trinucleotide context, the user may choose to set an additional parameter to normalize by the number of times each trinucleotide context is observed in the region sequenced. Trinucleotide counts for exome and genome data are provided in the package for this normalization. Alternatively, a user can also generate their own *T* data frame to use as input into deconstructSigs. A signatures matrix *S* of *k* rows and 96 columns is also defined, either calculated from published data [4] or provided by the user, where *k* is the number of supplied signatures. *S* consists of the fraction of times a mutation is seen in each of the 96 trinucleotide contexts for each signature *k*. Given these two inputs, *T* and *S*, deconstructSigs computes weights *W*_*i*_ (for each signature *i* from 1 to *k*) such that each signature has a weight. These weights are determined such that a reconstructed tumor sample matrix *R*, which is computed as *T-(SW)*, minimizes a given error threshold *e*.

This step is called with the function “whichSignatures” as shown below.

output.sigs = whichSignatures(tumor.ref = randomly.generated.tumors, signatures.ref = signatures.nature2013, sample.id = "1")

To determine the weights *W* that will best recreate *T*, an iterative approach is taken. First, we exclude any signatures containing a single trinucleotide context making up more than 20 % of the signature definition which is not present in *T*. This is done to account for the fact that some signatures are almost entirely characterized by mutations in specific trinucleotide contexts, and thus, without mutations found in those contexts, it is unlikely that that signature is active. From the remaining signatures, an initial mutational signature is chosen that most closely reflects the mutational profile of the given tumor sample by minimizing the sum-squared error (SSE) between the mutational profile of the tumor sample *T* and the mutational signature *S*_*i*_. The weights, *W*, are initialized such that the initial signature chosen, *S*_*i*_, is the only signature contributing to the reconstructed tumor mutational profile, thus being assigned a normalized weight of 1. A forward selection process subsequently determines, for each signature, the optimal weight that minimizes the SSE between the given tumor sample and the reconstructed tumor profile. From this set of possible weights, the weight corresponding to the signature that results in the overall lowest SSE is provided in *W*. This iterative process repeats until the difference between the SSE before and after the alteration of the weights matrix is less than an empirically chosen error threshold of 0.001.

**Fig. 1:**
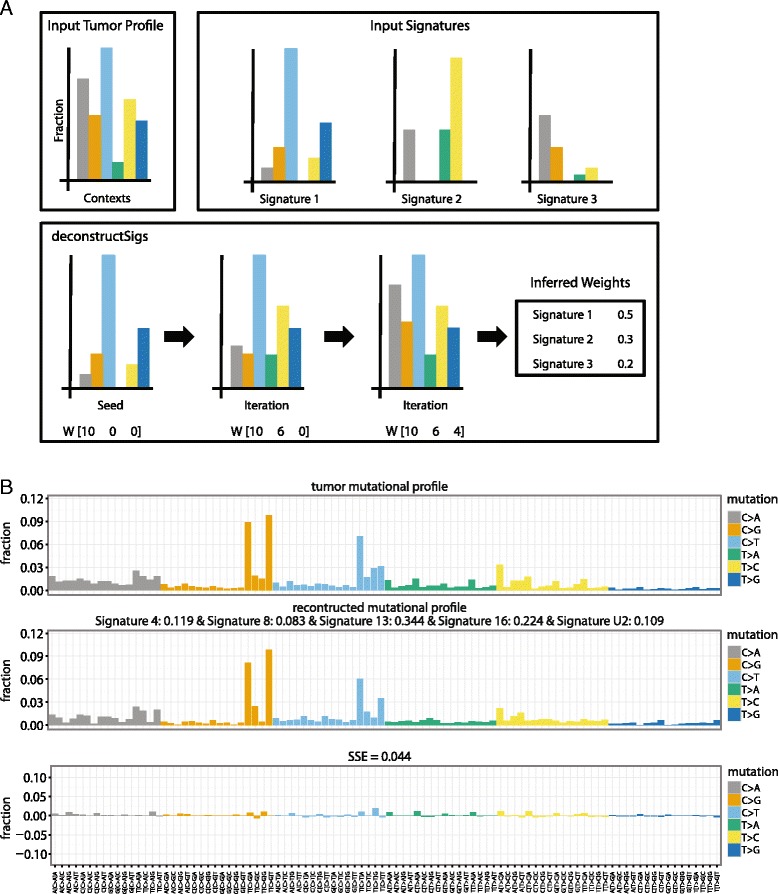
deconstructSigs workflow and output. **a** Given an input tumor profile and reference input signatures, deconstructSigs iteratively infers the weighted contributions of each reference signature until an empirically chosen error threshold is reached. **b** Example of the plot generated by the command ‘plotSignatures’. The *top panel* is the tumor mutational profile displaying the fraction of mutations found in each trinucleotide context, the *middle panel* is the reconstructed mutational profile created by multiplying the calculated weights by the signatures, and the *bottom panel* is the error between the tumor mutational profile and reconstructed mutational profile, with SSE annotated

Finally, the weights *W* are normalized between 0 and 1 and any signature with *W*_*i*_ < 6 % is excluded. This 6 % threshold was chosen by randomly generating tumors in silico whose mutational profiles were perturbed to be distant from the ideal theoretical sample. Initially, a set of 500 tumors representing a random combination of up to 10 of the published mutational signatures [4] was simulated. Because a tumor will never reflect a perfect combination of mutational signatures, these simulated tumors were perturbed by changing the calculated value at each trinucleotide context by up to ±5 %. These perturbed tumor samples were analyzed with deconstructSigs and the calculated weights were compared with the theoretical weights used to generate the set of simulated tumors. This analysis revealed that false positives had weights *W*_*i*_ routinely less than 6 % (Figure S1a in Additional file 1). Additionally, this 6 % cutoff only resulted in 38 instances where a signature was incorrectly excluded for a false negative rate of 1.4 % (Figure S1b in Additional file 1). A reconstructed tumor mutational profile *R* based on these final weights is determined as described above. A schematic of our deconstructSigs approach is outlined in Fig. 1a. To visualize the output, two plotting functions are available. The first compares the reconstructed tumor mutational profile with the original input tumor profile and is called using “plotSignatures”. Figure 1b shows an example of the generated plot. The second is a pie chart that shows the weights of each signature assigned in the sample and is called with “makePie”. They both use the output list given by “whichSignatures” as input.

## Results

### Comparison with WTSI Mutational Signature Framework

To investigate how deconstructSigs compares with the results generated by a user running the published WTSI Mutational Signature Framework on a new set of samples, we analyzed available data from The Cancer Genome Atlas (TCGA) on bladder urothelial carcinoma (BLCA), breast invasive carcinoma (BRCA), colon adenocarcinoma (COAD), glioblastoma multiforme (GBM), head and neck squamous cell carcinoma (HNSC), lung adenocarcinoma (LUAD), lung squamous cell carcinoma (LUSC), and skin cutaneous melanoma (SKCM) cancers (https://tcga-data.nci.nih.gov/tcga). The specific mutation files for these tumor types are detailed in Additional file 2. We first implemented the WTSI Mutational Signature Framework to independently extract signatures present in each cancer type. Twenty-six mutational signatures were extracted using the WTSI Mutational Signature Framework as described in McGranahan et al. [8]. For some TCGA cancer types, we found that the number of available samples was too low to achieve the resolution necessary to extract all the signatures previously associated with that cancer type. Reassuringly, the majority of signatures (20/26) extracted matched in profile those published by Alexandrov et al. [4] and were consistent with those original signatures. The age related signatures, 1A and 1B, were considered together as one signature. Two of the discordant signatures appeared to be a mix of two or three of the original signatures, highlighting again the importance of large sample numbers for accurately deconvoluting novel mutational signatures.

**Fig. 2:**
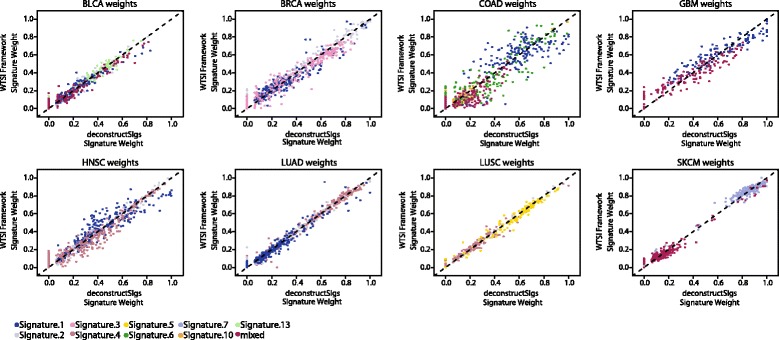
Comparison of signature contributions identified with deconstructSigs and WTSI Mutational Signature Framework. Scatterplots represent the relationship between the weighted proportions calculated using the WTSI Mutational Signature Framework method on a set of TCGA tumors and those inferred with deconstructSigs from the same set of patients. Each point plotted represents the weights assigned by both methods to one signature detected in a patient

All the newly extracted signatures were then used, as *S*, with deconstructSigs to analyze the same cohort of samples. This allowed for a direct comparison between the weighted proportions produced by the WTSI Mutational Signature Framework and those inferred with deconstructSigs. For every signature present in a given sample, we observed a statistically significant correlation (Additional file 3) between the contributions of that signature in the two independent methods (Fig. 2). These data indicated that we could consistently identify the signatures present in an individual tumor sample using deconstructSigs.

The SSE between the reconstructed mutational profile and the observed one, obtained by calculating the fraction of mutations present in each trinucleotide context, was consistently similar between the two approaches (Figure S2a in Additional file 4). The SSE calculated from both methods was higher in samples with a lower mutation count (Figure S2b in Additional file 4), highlighting the importance of having a sufficient number of mutations to identify and assign signatures characterized by 96-substitution classifications. This is of particular importance when the profile of the mutational signature is flat or without a strong peak at any of the trinucleotide contexts, as in these instances the mutational process could affect a greater number of trinucleotide contexts and a full profile would only be observed with sufficient mutations. Consequently, deconstructSigs warns the user if a sample contains fewer than 50 mutations.

**Fig. 3:**
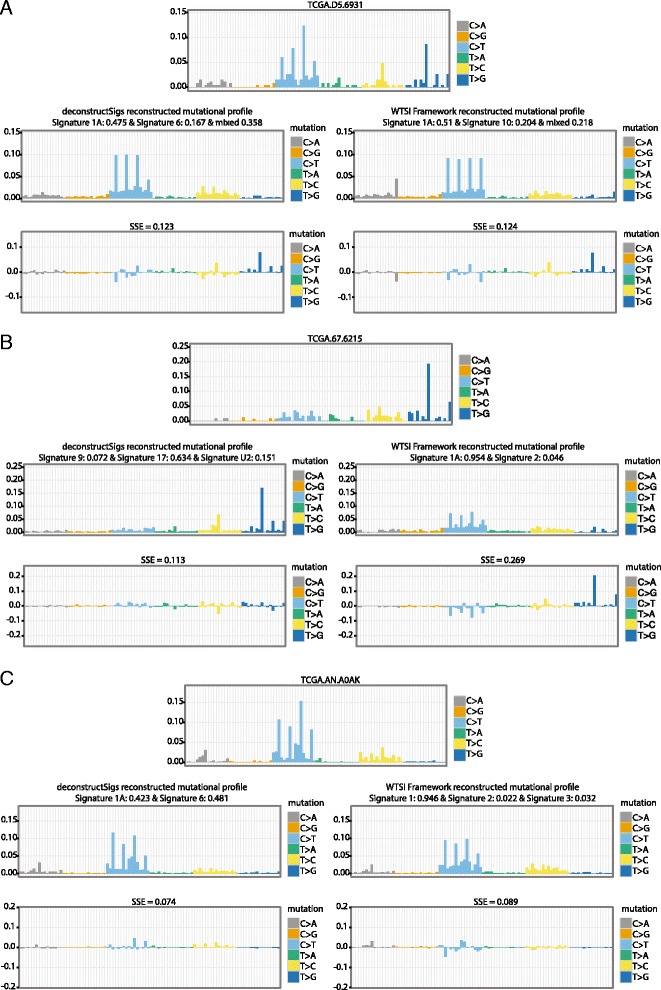
Specific TCGA patient examples. Comparison of tumor mutational profiles and reconstructed profiles output from deconstructSigs and WTSI Mutational Signature Framework. The reconstructed tumor profiles generated by using the signature weights assigned by the deconstructSigs method and the WTSI Mutational Signature Framework method are given for three tumor samples. **a** A signature associated with POLE hypermutation, signature 10, was identified in TCGA patient TCGA-D5-6931 using the WTSI Mutational Signature Framework (signature weight = 0.204) but not with deconstructSigs. However, a *POLE* exonuclease domain mutation was not observed in this patient. **b** The mutational profile of patient TCGA-67-6215 showed activity of Signature 17 but as this signature was not considered a possible signature extracted in the first step of the WTSI Mutational Signature Framework output, it was only called with deconstructSigs (signature weight = 0.634). **c** A signature associated with DNA mismatch repair deficiency, signature 6, was identified by deconstructSigs (signature weight = 0.481) in patient TCGA-AN-A0AK but was not identified by the WTSI Mutational Signature Framework. An *MSH6* frameshift mutation was identified in TCGA-AN-A0AK indicating the DNA mismatch repair deficiency signature identified is unlikely to be spurious

Additionally, the analysis uncovered a number of false positive mutational signatures that arise using the WTSI framework, whereby samples were erroneously classified as harboring a mutational signature. For instance, in a colorectal carcinoma (TCGA-D5-6931) the WTSI Mutational Signature Framework determined 20.4 % of the mutational signature present was associated with a POLE hyper-mutator phenotype. Nevertheless, visual inspection of the mutational profile of this tumor did not reveal the presence of a *POLE-*associated signature and no evidence existed for the somatic exonuclease domain mutations in *POLE* that produce this specific pattern of predominantly C > A mutations in a TpCpT context and C > T mutations in a TpCpG context (Fig. 3a).

To further explore the presence of both false positive and false negative mutational signatures using the WTSI mutational framework, we also repeated our analysis, expanding the signature space in which deconstructSigs could search. Thus, instead of using the newly extracted signatures by the WTSI mutational signature framework alone, we allowed deconstructSigs to use any of the original signatures [4]. For this analysis, we excluded any cancer types where the number of samples was originally too low such that extracting signatures using the WTSI Mutational Signature Framework method resulted in signatures that were a mixture of the published ones. In these instances a comparison between these ‘mixed’ signatures and the ability of deconstructSigs to identify them using the published signatures as a reference set could not be fairly made. The cancer types excluded were BLCA, COAD, GBM, and SKCM.

Re-extracted signatures through a separate iteration of the WTSI Mutational Signature Framework on new samples are always slightly different from the originally published ones, particularly when fewer samples are used for extraction as each signature is less well resolved. Thus, we did not expect to see a perfect correlation between the weights assigned by deconstructSigs using the original signatures as reference and the contributions found in our initial run of the WTSI Mutational Signature Framework (as was observed in Fig. 2). However, when we compared the weights assigned by deconstructSigs and the contributions by the WTSI Mutational Signature Framework, we saw a strong positive and statistically significant correlation for all signatures except signature 3 and signature 5 (Additional files 5 and 6). Notably, signatures 3 and 5 are characterized by relatively flat mutational profiles, exhibiting few distinguishing patterns of trinucleotide context.

Expanding the set of signatures also allowed us to identify outlier samples that appear to harbor a different set of mutational signatures compared with the rest of the cancer type or sample set they were analyzed with. Contributions can be missed by the WTSI Mutational Signature Framework if the signature is not prevalent enough in the sample set to be extracted as a separate entity. For instance, the LUAD sample TCGA-67-6215 has clear indications of signature 17 activity, a signature of unknown etiology (Fig. 3b). However, since signature 17 was not one of the signatures extracted through the implementation of the WTSI Mutational Signature Framework, it could not be identified as contributing to the observed mutational spectrum. Likewise, the deconstructSigs package assigned substantial contributions from the signature associated with DNA mismatch (MMR) repair deficiency (signature 6) to the BRCA samples TCGA-A8-A08F, TCGA-A8-A09Z, and TCGA-AN-A0AK (Fig. 3c), but signature 6 was not extracted through the implementation of the WTSI Mutational Signature Framework, nor was it associated with breast cancer in the work published in 2013 [4]. In two of these tumors, TCGA-A8-A09Z and TCGA-AN-A0AK, mutations in mismatch repair genes were evident (https://tcga-data.nci.nih.gov/tcga/). There was an *MLH1* nonstop mutation in TCGA-A8-A09Z and separate *MLH1* missense and splice site mutations, as well as an *MSH6* frameshift mutation in TCGA-AN-A0AK. Additionally, TCGA-A8-A09Z had a total of 1438 mutations with 253 small insertions or deletions and TCGA-AN-A0AK had a total of 1317 mutations with 352 small insertions or deletions, both indicative of a microsatellite instability high (MSIH) phenotype. The median number of mutations from the BRCA TCGA cohort is 38 and median number of insertions or deletions is 4.

Taken together, these results highlight the power of analyzing tumors on an individual basis, allowing the detection of mutational processes only active in a small subset of the samples considered.

### Identifying signatures in multi-region sequencing data

To further determine the performance of deconstructSigs, we examined a cohort of 19 tumors collected from six patients diagnosed with either LUAD or LUSC, with one tumor exhibiting an adenosquamous histological subtype. Multi-region whole-exome and/or genome sequencing was previously performed in these patients as described in de Bruin et al. [9] with mutations temporally dissected into trunk and branch mutations and the fraction of mutations occurring in each of the six possible base substitution classes previously established [9]. Given the limited number of tumor samples, the mutational catalogues from these samples were not amenable to a de novo analysis with the WTSI Mutational Signature Framework. We therefore used deconstructSigs with the 23 original signatures [4] to establish the contribution of individual mutational signatures to the samples.

**Fig. 4:**
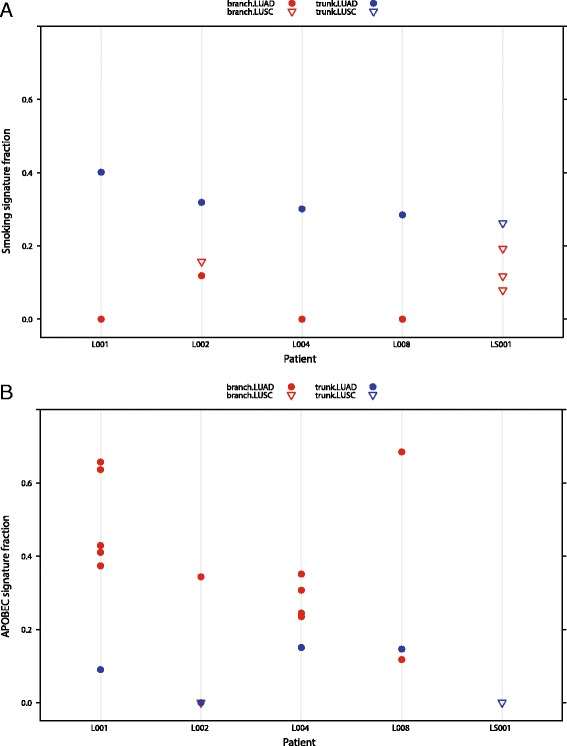
Temporal dissection of mutational processes. Mutations called from a cohort of five LUAD (*circles*) and LUSC (*triangles*) patients with multi-region sequencing were temporally dissected into trunk and branch mutations, described previously in [9]. One patient (L002) had a tumor exhibiting an adenosquamous histological subtype, with separate regions being of different histology. For each patient, the fraction of contribution of signatures associated with smoking (**a**) and APOBEC activity (**b**) was determined in the trunk and branch mutations. The smoking signature was seen at higher fractions in the trunk mutations (*blue*) than the branch mutations (*red*), whereas the signature of APOBEC activity was seen to contribute more to the LUAD branch mutations than LUAD trunk mutations or LUSC trunk or branch mutations

Whilst our original analysis utilized C > A mutations as a surrogate measure of a smoking signature resulting from tobacco exposure, deconstructSigs enabled us to refine this analysis. This new analysis allowed us to determine the specific contribution of signature 4, known to be associated with the number of smoking pack years [1, 4], rather than the more general C > A mutation class. Consistent with the previous analysis, we observed that the smoking associated signature (signature 4) was present at a higher fraction in the clonal mutations originating in the tumor trunk and found at a lower fraction in the subclonal mutations in the tumor branches (Fig. 4a). Indeed, in three of the five patients analyzed (L001, L004, and L008), the smoking signature was not assigned at all in the branches.

We next investigated the assigned weights of the APOBEC-associated signatures (signature 2 and signature 13) and found that in the LUAD subtypes, the signature was more pronounced in the branches than in the trunk (Fig. 4b), consistent with our published observations.

These results demonstrate the utility of deconstructSigs leveraging well-established signatures to determine the contribution of given mutational signatures to individual tumors, refining mutational processes present in cancer subclones.

### Exploring signatures in esophageal carcinoma

To further determine the utility of deconstructSigs, we applied our framework to each esophageal tumor (ESCA), both adenocarcinomas and squamous cell carcinomas from TCGA (https://tcga-data.nci.nih.gov/tcga). The mutation files were obtained from Broad Institute MAF dashboard (https://confluence.broadinstitute.org/display/GDAC/MAF+Dashboard). Notably, in the original publication by Alexandrov et al. [4], these two cancer types were considered in aggregate given the limited number of samples available. Using deconstructSigs, it was possible to directly compare esophageal tumors originating from these different cell types. In addition, in order to shed light on both the prevalence of mutational processes and their dynamics during tumor evolution, we also applied the deconstructSigs package to temporally dissected mutations (Additional file 7), according to published methods [8]. This allowed us to identify different mutational processes contributing to early and late/subclonal mutations.

**Fig. 5:**
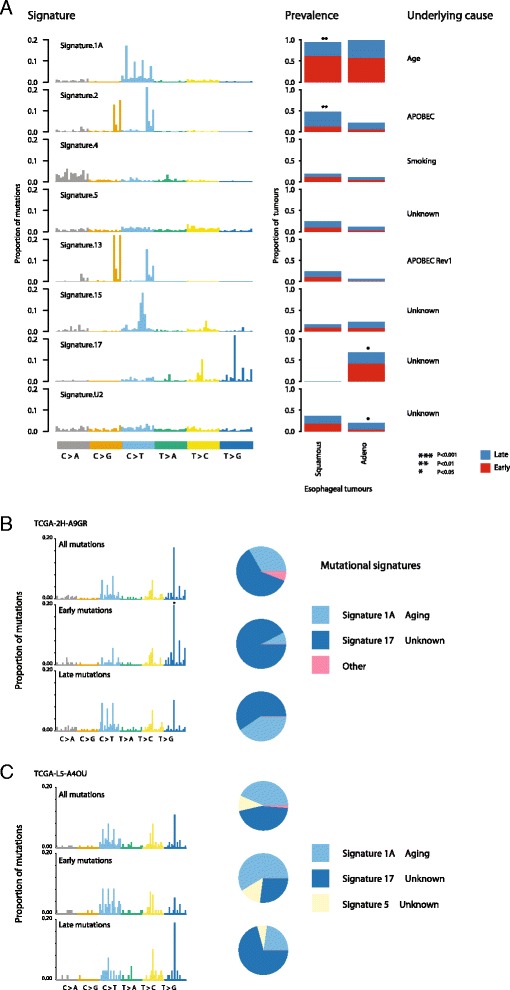
Signatures present in esophageal carcinoma. **a** The signatures identified in a cohort of ESCA tumors by deconstructSigs, using the input reference signatures from [4]. The prevalence, defined as the fraction of patients the signature was detected in, is plotted for each mutational signature identified, and the proportion of patients with a higher fraction of early (*red*) or late (*blue*) mutations corresponding to that signature is shown. **b**, **c** A specific analysis of two esophageal adenocarcinomas exhibiting signs of signature 17 activity. The mutational profiles are given for all the mutations identified in both tumors, as well as the mutations classified as early or late. Signature 17 was identified as the largest contributor to the early mutations of patient TCGA-2H-A9GR (**b**) whereas it was identified as the contributing to the generation of the majority of late mutations in patient TCGA-L5-A4OU (**c**)

In total, across these 169 esophageal tumors, eight mutational signatures were evident, many of which were found to contribute to varying degrees during different periods of the disease course. The most prevalent signature in both esophageal adenocarcinoma and squamous cell carcinomas, signature 1A, which likely reflects spontaneous deamination of methylated cytosines, was detected in almost all ESCA tumors studied. Interestingly, this signature was found to contribute significantly more to early mutations compared with late mutations in esophageal squamous cell carcinoma but not in esophageal adenocarcinoma (ESCA squamous, *p* value = 0.006; ESCA adeno, *p* value = 0.716; Fig. 5a).

Signature 2, likely reflecting APOBEC-mediated mutagenesis, was identified as contributing significantly more in squamous cell compared with adenocarcinoma esophageal cancers (*p* value = 0.01). Further, in squamous cell tumors, APOBEC-mediated mutagenesis was found frequently to be a late event, reflecting similar findings in lung adenocarcinomas, head-and-neck tumors, and estrogen receptor-negative breast cancers [8] (*p* value = 0.03; Fig. 5a). Conversely, no significant trend was observed in esophageal adenocarcinomas.

Another clear difference between esophageal tumors of these two cell types was the presence of signature 17, a signature of unknown etiology, exclusively in adenocarcinomas. Indeed, over 50 % of esophageal adenocarcinomas were found to exhibit an enrichment for T > G and T > C mutations at CpTpT sites. The majority of these tumors (65 %) showed a tendency for signature 17 to be an early event, often being replaced by signature 1A. For example, in one such tumor (TCGA-2H-A9GR), early mutations were almost exclusively characterized as signature 17 (90.4 %), while later arising mutations were characterized by an increase in signature 1A at the expense of signature 17. These data corroborate recent findings using multi-region sequencing [10]. Nevertheless, we also identified a subset of esophageal adenocarcinomas in which signature 17 increased in prevalence over time. For example, TCGA-L5-A4OU exhibited a marked increase in signature 17 among its late mutations, accompanied by a decrease in signature 1A and signature 5 (Fig. 5b, c).

Taken together, these results illustrate the utility of deconstructSigs to reveal the mutational processes in individual cancers, enabling comparisons of distinct histologies within tumor types and the elucidation of the dynamics of these mutational signatures over time.

## Conclusions

Here we present a computational approach, deconstructSigs, which determines the composition of a given set of mutational signatures in individual tumor specimens. We have demonstrated that through using deconstructSigs we can consistently identify the same signatures of mutational processes active in a single tumor sample compared with the analysis of an entire sample set using the WTSI Mutational Signature Framework [8]. We have also shown some of the potential benefits of analyzing samples on an individual basis, as a user can both detect mutational processes active in only a small number of samples and investigate well-established signatures without having to consider or compile a large sample set. Furthermore, we utilized this approach to consider how the activity of mutational processes changes in individual tumors over time.

Due to the recurrent nature of many mutational signatures, present across multiple tumor types, there is much interest in identifying these existing signatures in further tumor samples. The input signature set, which by default is the set of already published signatures, can also be a user-defined parameter, so it is possible for it to be adapted as mutational signatures are further identified and refined through large-scale genomics analyses. For instance, 30 signatures are now identified by the Wellcome Trust Sanger Institute (http://cancer.sanger.ac.uk/cosmic/signatures), some of which are identified in tumor types not considered here, such as stomach cancer, kidney clear cell carcinoma, and Hodgkin’s lymphoma. In future studies and as new signatures are identified, these signatures could be included in the input signatures set by the user. Thus, we anticipate that deconstructSigs will complement other efforts to define and identify mutational processes.

Finally, as the sequencing of individual tumors becomes increasingly common in a clinical setting, we expect that the ability to determine contributions of specific mutational processes within single samples will allow for novel insights, revealing cancer vulnerabilities that may guide clinical decision-making on a case-by-case basis. It will be possible to identify potential environmental exposures within individual tumors, which may provide utility within a medico-legal setting. Finally, this tool will enable the impact that previous therapies have had on shaping the cancer genome to be defined and further our understanding into the dynamic evolutionary processes between primary and metastatic sites within individual patients.

## Availability of data and materials

The results published here are in part based on data generated by TCGA project established by the National Cancer Institute and National Human Genome Research Institute. The data were retrieved through dbGaP (Database of Genotypes and Phenotypes) authorization (accession number phs000178.v9.p8). Information about TCGA and the investigators and institutions that constitute the TCGA research network can be found at http://cancergenome.nih.gov/.

The multiregion sequencing data used can be found in the European Genome-Phenome Archive (EGA, https://www.ebi.ac.uk/ega/), under accession numbers EGAS00001000840 and EGAS00001000809. The deconstructSigs package (v1.6.0) is available on the Comprehensive R Archive Network (CRAN, https://cran.r-project.org/) under a GPL-2 license. It has also been deposited to Zenodo (https://zenodo.org/) with a DOI (http://dx.doi.org/10.5281/zenodo.45311).

## Ethics approval

Ethics approval was not needed for this study.

## Additional files

Additional file 1: Figure S1. Weights assigned to false positives and false negatives in a randomly generated tumor cohort. A random cohort of 500 tumors containing 2646 total signatures was generated with known signature contributions of the published signatures. This cohort was subjected to up to a ±5 % random perturbation to more accurately reflect a ‘non-perfect’ theoretical tumor sample. Running deconstructSigs on these simulated tumor samples resulted in some outputs containing false positives, where a signature was erroneously identified as contributing to the sample (**a**). The weights assigned to these false positive results were seen almost uniformly have been under 6 % for each signature (marked at the *red line*). **b** False negatives, where a signature was erroneously rejected as contributing to the sample, occurred 38 times from the analysis of the randomly simulated tumors for a false negative rate of 1.4 %. The weights of all of the false negatives were under 6 %, indicating that the use to this cutoff does not increase the tendency of deconstructSigs to call false negatives. (PDF 111 kb) Additional file 2:
**Supplementary methods, containing the specific TCGA mutation files used in all analyses.** (DOCX 86 kb) Additional file 3: Table S1. The mutational signature contribution to each TCGA tumor sample studied as determined by deconstructSigs and WTSI Mutational Signature Framework. Signatures used as input to deconstructSigs were limited to those extracted using the WTSI Mutational Signature Framework approach. (TXT 464 kb) Additional file 4: Figure S2. Comparison of the SSE between deconstructSigs and WTSI Mutational Signatures Framework. **a** SSEs between the input tumor mutational profile and reconstructed mutational profile were calculated for each TCGA tumor analyzed. The calculated SSEs from using the WTSI Mutational Signatures Framework were compared with those from using deconstructSigs. Each point represents the SSE as calculated through use of the signature weights assigned by the WTSI Mutational Signatures Framework and the SSE as calculated through the use of the signature weights assigned by deconstructSigs. The SSE is consistent between the two approaches. **b** Relationship between SSE and overall mutation count. As the mutation count of the tumor sample increases, the calculated SSE decreases. (PDF 632 kb) Additional file 5: Figure S3. Comparison of signature contributions between deconstructSigs and WTSI Mutational Signature Framework using reference signatures. Cancer types with unambiguous signatures extracted using WTSI Mutational Signatures Framework [8] were re-analyzed with deconstructSigs and allowed to use any of the originally published signatures [4]. For the signatures that were extracted using WTSI Mutational Signature Framework, a comparison between the weights assigned by deconstructSigs and those originally calculated by WTSI Mutational Signatures Framework is plotted. A table of the values of all weights assigned by deconstructSigs can be found in Table S2 (Additional file 6). (PDF 321 kb) Additional file 6: Table S2. The mutational signature contribution to each TCGA tumor sample studied as determined by deconstructSigs and WTSI Mutational Signature Framework. The full published set of mutational signatures [4] was allowed to be used as input to deconstructSigs. Tumor types considered were limited to those where only unambiguous signatures could be extracted using the WTSI Mutational Signature Framework to allow for direct comparison with the input reference signature set. (TXT 359 kb) Additional file 7: Table S3. Mutations from TCGA ESCA cohort and their timing (early/late). (TXT 811 kb)
