# Supplementary figures and images for: deconstructSigs: delineating mutational processes in single tumors distinguishes DNA repair deficiencies and patterns of carcinoma evolution
Source: Genome Biol. 2016 Feb 22;17:31. doi: 10.1186/s13059-016-0893-4 (PMC4762164; doi:10.1186/s13059-016-0893-4)

Supplementary Figure 1

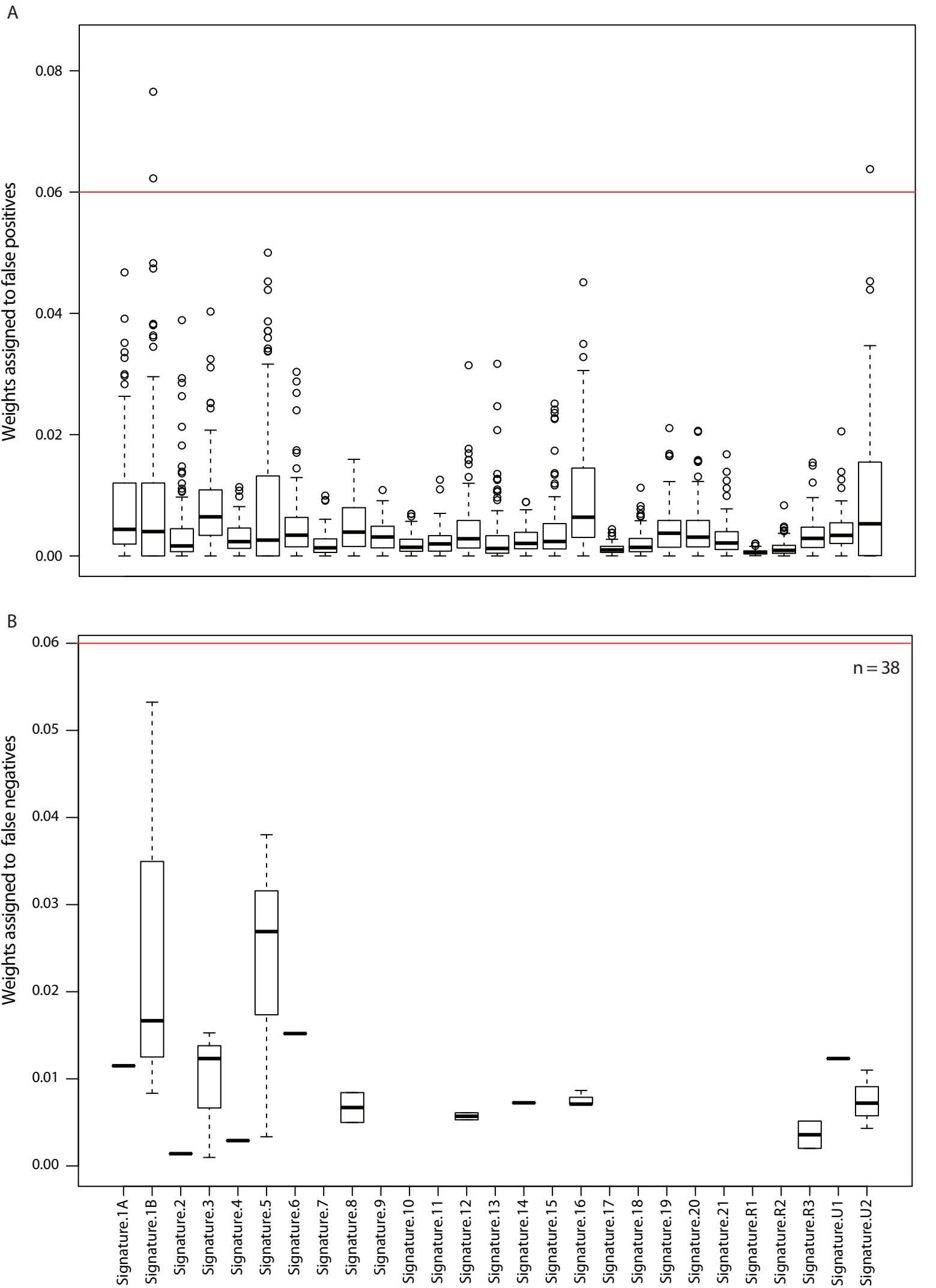

Supplement: Additional file 1: Figure S1. — Weights assigned to false positives and false negatives in a randomly generated tumor cohort. A random cohort of 500 tumors containing 2646 total signatures was generated with known signature contributions of the published signatures. This cohort was subjected to up to a ±5 % random perturbation to more accurately reflect a ‘non-perfect’ theoretical tumor sample. Running deconstructSigs on these simulated tumor samples resulted in some outputs containing false positives, where a signature was erroneously identified as contributing to the sample (a). The weights assigned to these false positive results were seen almost uniformly have been under 6 % for each signature (marked at the red line). b False negatives, where a signature was erroneously rejected as contributing to the sample, occurred 38 times from the analysis of the randomly simulated tumors for a false negative rate of 1.4 %. The weights of all of the false negatives were under 6 %, indicating that the use to this cutoff does not increase the tendency of deconstructSigs to call false negatives. (PDF 111 kb) [file 13059_2016_893_MOESM1_ESM.pdf]

Supplementary Figure 2

A

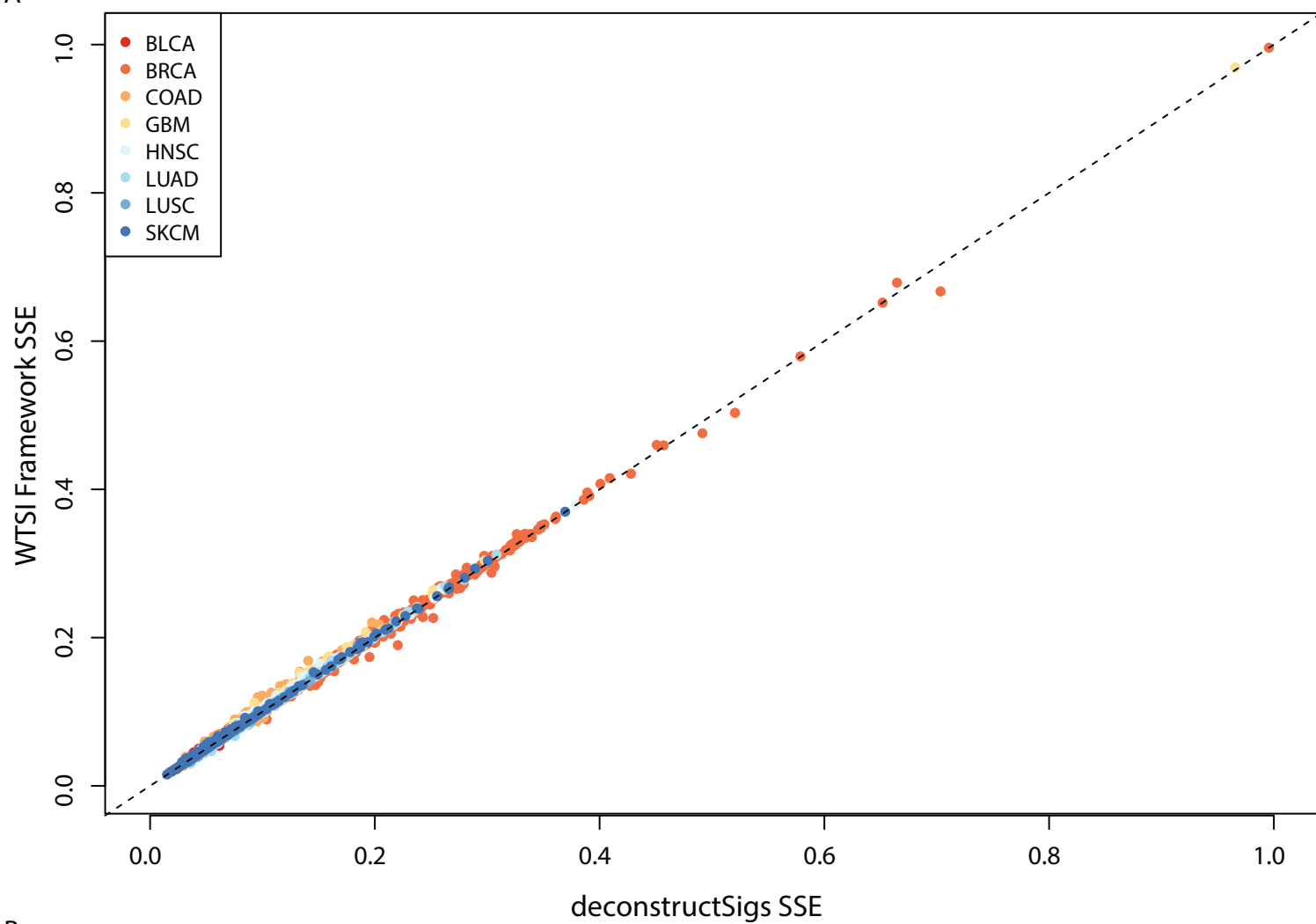

B

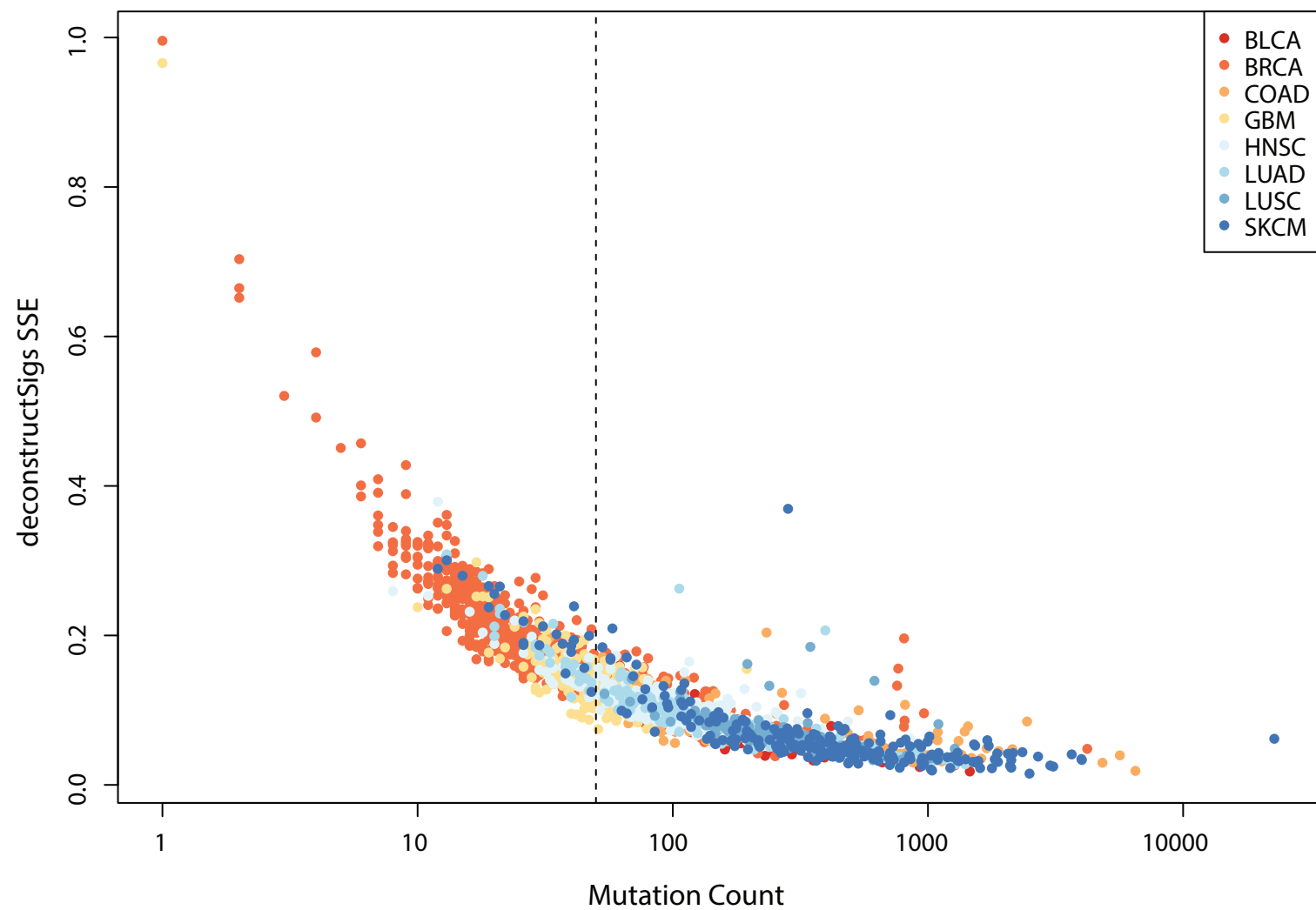

Supplement: Additional file 4: Figure S2. — Comparison of the SSE between deconstructSigs and WTSI Mutational Signatures Framework. a SSEs between the input tumor mutational profile and reconstructed mutational profile were calculated for each TCGA tumor analyzed. The calculated SSEs from using the WTSI Mutational Signatures Framework were compared with those from using deconstructSigs. Each point represents the SSE as calculated through use of the signature weights assigned by the WTSI Mutational Signatures Framework and the SSE as calculated through the use of the signature weights assigned by deconstructSigs. The SSE is consistent between the two approaches. b Relationship between SSE and overall mutation count. As the mutation count of the tumor sample increases, the calculated SSE decreases. (PDF 632 kb) [file 13059_2016_893_MOESM4_ESM.pdf]
