# Supplementary material for: deconstructSigs: delineating mutational processes in single tumors distinguishes DNA repair deficiencies and patterns of carcinoma evolution
Source: Genome Biol. 2016 Feb 22;17:31. doi: 10.1186/s13059-016-0893-4 (PMC4762164; doi:10.1186/s13059-016-0893-4)
Supplement: Additional file 2: — Supplementary methods, containing the specific TCGA mutation files used in all analyses. (DOCX 86 kb) [file 13059_2016_893_MOESM2_ESM.docx]

**Supplementary Methods**

TCGA data files

TCGA data was obtained from Broad Institute MAF dashboard (<https://confluence.broadinsttitute.org/display/GDAC/MAF+Dashboard>) as detailed:

BLCA:

- PR_TCGA_BLCA_PAIR_Capture_All_Pairs_QCPASS_v3.aggregated.capture.tcga.uuid.somatic.maf (center: broad.mit.edu, archive version: 0.3.0)

BRCA:

- genome.wustl.edu_BRCA.IlluminaGA_DNASeq.Level_2.1.1.0.curated.somatic.maf (center: genome.wustl.edu, archive version: 1.1.0)

COAD:

- hgsc.bcm.edu_COAD.IlluminaGA_DNASeq.1.somatic.maf (center: hgsc.bcm.edu, archive version: 1.5.0)

ESCA:

- An_TCGA_ESCA_External_capture_All_Pairs.aggregated.capture.tcga.uuid.automated.somatic.maf (center: broad.mit.edu, archive version: 1.0.0)

GBM:

- step4_gbm_liftover.aggregated.capture.tcga.uuid.maf2.4.migrated.somatic.maf (center: broad.mit.edu, archive version: 1.4.0)

HNSC:

- PR_TCGA_HNSC_PAIR_Capture_All_Pairs_QCPASS_v2.aggregated.capture.tcga.uuid.somatic.maf (center: broad.mit.edu, archive version: 0.2.0)

LUAD:

- PR_TCGA_LUAD_PAIR_Capture_All_Pairs_QCPASS_v4.aggregated.capture.tcga.uuid.automated.somatic.maf (center: broad.mit.edu, archive version: 1.5.0)

LUSC:

- LUSC_Paper_v8.aggregated.tcga.somatic.maf (center: broad.mit.edu, archive version: 1.5.0)

SKCM:

- PR_TCGA_SKCM_PAIR_Capture_All_Pairs_QCPASS_v4.aggregated.capture.tcga.uuid.automated.somatic.maf (center: broad.mit.edu, archive version: 1.4.0)
