# Supplementary material for: deconstructSigs: delineating mutational processes in single tumors distinguishes DNA repair deficiencies and patterns of carcinoma evolution
Source: Genome Biol. 2016 Feb 22;17:31. doi: 10.1186/s13059-016-0893-4 (PMC4762164; doi:10.1186/s13059-016-0893-4)

Supplementary Figure 3

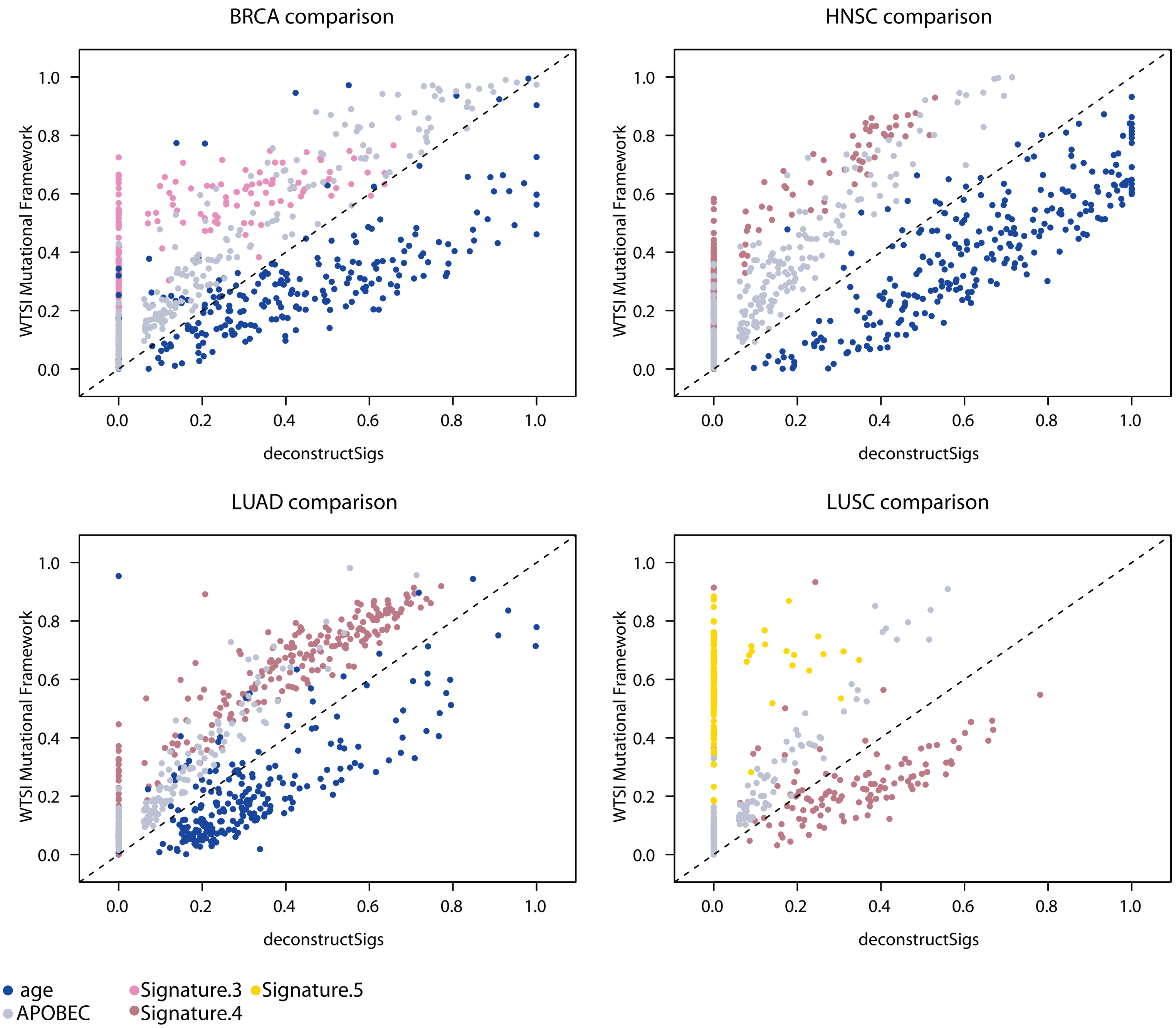

Supplement: Additional file 5: Figure S3. — Comparison of signature contributions between deconstructSigs and WTSI Mutational Signature Framework using reference signatures. Cancer types with unambiguous signatures extracted using WTSI Mutational Signatures Framework [8] were re-analyzed with deconstructSigs and allowed to use any of the originally published signatures [4]. For the signatures that were extracted using WTSI Mutational Signature Framework, a comparison between the weights assigned by deconstructSigs and those originally calculated by WTSI Mutational Signatures Framework is plotted. A table of the values of all weights assigned by deconstructSigs can be found in Table S2 (Additional file 6). (PDF 321 kb) [file 13059_2016_893_MOESM5_ESM.pdf]
